# The Medical Convention

**Published:** 1860-10

**Authors:** 


*The Medical Convention.*—In June last, the American Medical Association passed a resolution recommending medical schools to reject all students not possessing a classical education. Not wishing to enter upon the merits of the matter, we would have preferred a resolution recommending the schools to be more exacting in their requirements from candidates for the M. D. What is wanted is, that this suffix to a name, should stand as an incontestable fact, that at the time of its acquisition, the candidate at least was qualified to aid man in his struggle for life. The best preliminary education will not fit all men to become doctors. This can only be known when he places himself before an examining body, who themselves being qualified to judge, make just, exact, and conscientious requirements of indubitable proof. This would remedy, to a great extent, the evil such a resolution is intended to arrest. High merit, alone, should carry a candidate through a trial which should be deep and earnest, looking to the cause of humanity, which often rests so completely under the charge of the physician. Abolish competition in schools and sympathy in men, and our present system is wise enough. B.

